# Association between Pre-Pregnancy BMI and Inflammatory Profile Trajectories during Pregnancy and Postpartum in Brazilian Women with Periodontitis: The IMPROVE Trial

**DOI:** 10.3390/ijerph19052705

**Published:** 2022-02-25

**Authors:** Danilo Dias Santana, Gilberto Kac, Pedro Paulo Teixeira dos Santos, Thainá Castro da Silva, Camila Benaim, Paula Guedes Cocate, Maria Beatriz Trindade de Castro, Berit Lilienthal Heitmann, Amanda Rodrigues Amorim Adegboye

**Affiliations:** 1 Nutritional Epidemiology Observatory, Department of Social and Applied Nutrition, Institute of Nutrition Josué de Castro, Federal University of Rio de Janeiro, Rio de Janeiro 21941-902, Brazil; dias.danilo@hotmail.com (D.D.S.); gilberto.kac@gmail.com (G.K.); dr2p@hotmail.com (P.P.T.d.S.); thainacastro6@gmail.com (T.C.d.S.); camilabenaimnutri@gmail.com (C.B.); mbtcastro@gmail.com (M.B.T.d.C.); 2 School of Physical Education and Sports, Federal University of Rio de Janeiro, Rio de Janeiro 21941-599, Brazil; paulacocate@gmail.com; 3 Research Unit for Dietary Studies, The Parker Institute, Bispebjerg and Frederiksberg Hospital, 2000 Frederiksberg, Denmark; berit.lilienthal.heitmann@regionh.dk; 4 Section for General Practise, Department of Public Health, University of Copenhagen, 1353 Copenhagen, Denmark; 5 Centre for Healthcare Research, Faculty of Health and Life Sciences, School of Nursing, Midwifery and Health, Coventry University, Priory Street, Coventry CV1 5FB, UK

**Keywords:** pre-pregnancy BMI, inflammatory markers, C-reactive protein, interleukins, predictors, pregnant women, periodontitis

## Abstract

This study aimed to explore the association between pre-pregnancy BMI and longitudinal changes in inflammatory markers from the second trimester of pregnancy to 6–8 weeks postpartum in women with periodontitis. This is a secondary exploratory analysis of 68 women who took part in a feasibility clinical trial in Rio de Janeiro, Brazil. Inflammatory markers included C-reactive protein (CRP), interleukin-6 (IL-6), interleukin-10 (IL-10), and matrix metalloproteinase-9 (MMP-9) blood concentrations at 11–22 (T0) and 30–36 gestational weeks (T1), and 6–8 weeks postpartum (T3). Longitudinal generalised linear mixed-effects models were used to identify possible associations between pre-pregnancy BMI and changes in concentrations of inflammatory markers. Pre-pregnancy excess weight (β = 4.39; 95% CI, 2.12–6.65) was significantly associated with increased CRP levels from pregnancy to postpartum. There were no significant associations between pre-pregnancy BMI and longitudinal changes in IL-6, IL-10 and MMP-9. Our findings provide evidence that a higher pre-pregnancy BMI may lead to increases in CRP levels during pregnancy in women with periodontitis, irrespective of the severity of clinical periodontal parameters. Further studies need to investigate if predictors of changes in inflammatory markers can be used as prognostic factors for gestational outcomes.

## 1. Introduction

Periodontitis, a bacterial infection of the gums and dental supporting structures, is common in women of reproductive age [1]. It tends to worsen during pregnancy due to physiological hormonal changes [2]. Observational studies have found associations between periodontitis and adverse pregnancy outcomes, including preterm birth [3], preeclampsia [4], miscarriage [5], and post-cesarean endometritis [6]. Periodontitis is also associated with elevated systemic inflammatory biomarkers [7,8]. The bacterial activity stimulates an immunological cell response with the release of cytokines which upon entering the bloodstream can reach the uterus and pass through the foetal placental barrier [9,10] and may directly affect foetal neuronal development, as demonstrated in animal studies [11].

Changes in C-reactive protein (CRP) are the most commonly investigated inflammatory biomarker during pregnancy, but only a few studies have reported on the longitudinal changes of interleukin-6 (IL-6), interleukin-10 (IL-10), or matrix metalloproteinase 9 (MMP-9). CRP is an acute-phase inflammatory biomarker primarily stimulated by IL-6 as a reaction of tissues to infection or inflammation [12]. The role of IL-6 during chronic inflammation is still not well understood; however, it is known that in patients with inflammation, such as periodontitis, circulating levels of IL-6 are often increased [13]. The MMP-9 is also involved in the pathological regulation processes, including inflammation and innate immune defense [14]. It has been demonstrated that circulating MMP-9 levels are higher in people with diabetes mellitus, cardiovascular diseases and cancer [15]. In contrast, IL-10 is an anti-inflammatory cytokine with an important role in maternal immune tolerance of the foetus in normal pregnancy [16,17].

Evidence suggests an association between these pro- and anti-inflammatory markers and gestational outcomes, including preterm and low birth weight neonates in healthy pregnant women [18] and among those with periodontitis [19]. In a prospective study conducted in Brazil, pregnant women in the higher tertile of the CRP trajectory gave birth to infants with a lower mean birth weight Z-score than pregnant women in the first/second CRP tertiles [20].

It has been observed that pregnant women with obesity have a more pronounced increase in CRP concentrations during gestation than pregnant women with normal weight [21]. Furthermore, pre-pregnancy obesity and several lifestyle factors, including a diet rich in meat and processed foods, correlate directly with high concentrations of circulating inflammatory biomarkers, such as CRP and IL-6 [22,23].

As studies on predictors of changes in inflammatory profile during pregnancy and early postpartum among women with periodontitis are scarce, and such studies are essential to further our understanding of the relationship between modifiable risk factors and changes in inflammatory markers and gestational outcomes, we aimed to describe the inflammatory profile trajectory during pregnancy and early postpartum and explore its potential association with pre-pregnancy BMI among pregnant/postpartum women with periodontitis. The exploratory hypothesis was that pre-pregnancy BMI would be independently associated with changes during pregnancy and early postpartum in inflammatory markers (CRP, IL-6, and MMP-9) and anti-inflammatory cytokine (IL-10) concentrations in pregnant women with periodontitis.

## 2. Methods and Materials

### 2.1. Study Design

This study was an offshoot of the main project entitled “Calcium and vitamin D supplementation and/or periodontal therapy in treating periodontitis among Brazilian pregnant women (the IMPROVE trial)”. The IMPROVE was a feasibility randomised controlled trial with a 2 × 2 factorial design and a parallel process evaluation. The current study consists of a secondary and exploratory analysis of the IMPROVE feasibility trial. Thus, in this study, we used the data as a cohort study rather than an intervention.

Pregnant women with periodontitis were recruited and randomly allocated into four groups: (1) fortified milk with vitamin D and calcium plus non-surgical periodontal treatment (PT) during pregnancy, (2) plain milk plus PT during pregnancy, (3) fortified milk with vitamin D and calcium plus PT after delivery and (4) plain milk plus PT after delivery. Pregnant women were allocated into intervention groups by block permutation randomisation (Sealed Envelope Ltd., https://www.sealedenvelope.com (accessed on 25 February 2019). More details can be found elsewhere [24].

### 2.2. Recruitment

Participants were recruited from May 2017 to May 2018 with follow-up until February 2019. The research was carried out in Duque de Caxias Municipal Health Center, Rio de Janeiro/Brazil.

Duque de Caxias is a city situated in a low socioeconomic area in Rio de Janeiro state with approximately 900,000 inhabitants [25]. In 2012 (last available data), this city presented a neonatal mortality rate of 12.1 per 1000 live births, 0.7% of maternal deaths, and 20.4% of families were covered by the family health strategy (FHS) [26]. FHS is targeted at people living in deprivation and whose nutritional status is impacted by social and economic factors [27].

In the first prenatal visit, pregnant women were invited to answer a checklist for eligibility. The inclusion criteria were age ≥ 18 years at the time of recruitment; <20 weeks of gestation at first prenatal visit; having both the physical and cognitive ability to complete an interview and oral examination and being willing to take part, which includes providing blood samples. The exclusion criteria were being diagnosed with psychosis, diabetes before pregnancy, thyroid disease, or any disorder implicated in vitamin D hypersensitivity (e.g., sarcoidosis and other lymphomatous disorders); having extensive dental cavities (crowns of several teeth destroyed by caries), and loss of tooth structure or use of fixed dental braces.

**Figure 1:**
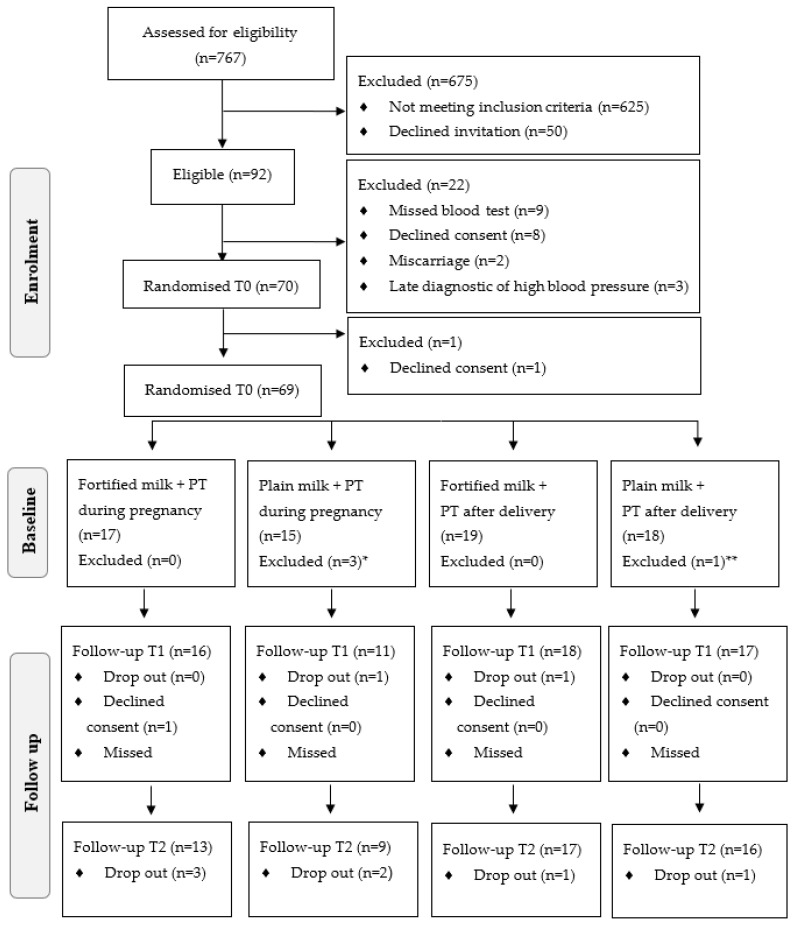
Study flowchart of enrolment, allocation, and follow-up of the pregnant women. PT: Periodontal treatment. * Did not start periodontal treatment (*n* = 1) or milk consumption (*n* = 1) or both (*n* = 1). ** did not start milk consumption (*n* = 1).

Those preliminarily eligible and who tested negative for syphilis and HIV were then invited to book a dental examination. Women who screened positive for periodontitis (≥1 tooth with at least one site with ≥4 mm of clinical attachment loss (CAL) and the presence of bleeding on probing (BOP)) were provided with an informed consent form and were subsequently included in the study. More information about patients’ recruitment is described elsewhere [24,28]. Of the 767 pregnant women assessed for eligibility, 92 met the initial eligibility criteria and were diagnosed with periodontitis. Eight removed their consent, nine did not attend the baseline blood test, two suffered a miscarriage, and three were diagnosed with hypertension. Of the 70 women randomised, 69 participated in the first visit, but one withdrew the consent (Figure 1) and one was excluded from the analysis due to lack of information about pre-pregnancy BMI. The final study population included 68 women.

All study participants were advised not to change their routine physical activity or consume any other supplements apart from the ones provided by the health centre (usually 400 μg/d folic acid and 60 mg/d ferrous sulphate). More details can be found in Adegboye et al. [28].

### 2.3. Oral Health Examination

Following the initial oral health screening during the recruitment stage, women with confirmed periodontitis who agreed to participate in the study underwent a full-mouth periodontal examination at baseline (T0—between 11 and 22 gestational weeks) and postpartum (T2—6 and 8 weeks after delivery), regardless of group allocation. Clinical periodontal parameters were not collected at the third trimester (T1). Periodontal examination was performed at six sites per tooth, including mesiobuccal, mid-buccal, distobuccal, mesiolingual, mid-lingual, and distolingual sites, using colour-coded North Carolina periodontal probes and a dental mirror. No X-ray was taken.

After participants were randomised into four study groups (Figure 1), those in the early PT group immediately underwent conventional non-surgical PT throughout pregnancy up to childbirth, consisting of prophylactic dental polishing to remove the bacterial film, scaling and root planning, as necessary. The number of therapy sessions varied according to disease severity, with a maximum of five sessions per participant. The treatment was performed by a trained dentist who was not involved in dental screening. Participants allocated to the late PT group started the treatment after childbirth as part of the routine dental care in the health centre. X-rays and anaesthesia were not used during the procedures.

Additionally, bi-monthly maintenance periodontal examinations were performed in those who had completed the PT during pregnancy. A tailored form was developed to register the data on gingival BOP (BOP), pocket depth, CAL, and dental mobility. Calibrated and trained dentists performed oral examinations and PT. The dentists calibrated their probing force using a scale before all examinations to ensure that an adequate pressure of approximately 20 g was applied [29]. The dentist who performed the periodontal examination was blinded regarding the participants’ group allocation.

### 2.4. Data Collection

All randomised women were examined and answered questionnaires during the period between the second (T0—before randomization) and third (T1—30–36 gestational weeks) trimesters of gestation and early postpartum (T2).

Gestational age (weeks) was calculated based on data from the first ultrasonography performed before the 20th week of gestation. Maternal blood samples (for analysis of biochemical markers) were collected in the morning after a 12-h overnight fast in all three-time points (T0, T1, and T2). The blood samples were centrifuged and stored in a freezer with a temperature of −80 °C until analysis. Fasting glucose levels (serum) were estimated by the enzymatic colorimetric method and insulin (serum) levels by chemiluminescence. Both markers were analysed by the Laboratory of Clinical Analysis and Pathology LTDA-SEPAC (Rio de Janeiro, Brazil).

Pregnant women answered structured questionnaires about sociodemographic characteristics and lifestyle, including age (years), years of education (years), monthly per capita income (USD), marital status (living with partner/others), self-reported skin colour (white/others), smoking habits (no/current smoker), alcohol consumption (no/yes), sun exposure (<30 min/day/>30 min/day) and maternal conditions such as parity. Parity was used as a binary variable (no previous delivery and one or more previous deliveries) in descriptive analyses and as a continuous variable (number of parturitions) in the longitudinal models. Gestational weight gain (GWG) (kg) was calculated as the difference between the weight measured in the third trimester and the self-reported pre-pregnancy weight [30].

Dietary intake was estimated through a validated semi-quantitative food frequency questionnaire (FFQ) [31] during the first follow-up visit. FFQ items were transformed into daily portions according to the daily frequency, and portion size intakes using a method previously described [32]. The Brazilian Food Composition tables [33,34] were used to determine the composition of foods. The US Department of Agriculture nutrient database [35] was used for foods lacking the Brazilian food composition database. These analyses were used to determine the total energy (kcal/d), calcium (mg/d), and vitamin D (mcg/d) intake.

Furthermore, data from 24 food nutrients (kcal, protein, fiber, saturated fat, monounsaturated fat, polyunsaturated fat, cholesterol, iron, thiamine (B1), riboflavin (B2), niacin, pyridoxine (B6), vitamin A (retinol), beta-carotene (RE), beta-carotene (RAE), folic acid, vitamin C and D, vitamin E, omega 3 (18:3n-3 (18:3)), omega 6 (18:2n-6 (18:2)), calcium, zinc and magnesium) were used to calculate the dietary inflammatory index (DII) score, according to Shivappa et al. [36]. For example, total fat, saturated fat, and cholesterol contribute negatively to calculating the overall DII score. In contrast, polyunsaturated fat, fibre, beta-carotene, and other antioxidants contribute positively to calculating the overall DII score. The main objective of calculating this score was to provide a tool to categorise diets on a continuum from maximally anti-inflammatory (negative values) to maximally pro-inflammatory (positive values) [37].

We used the Pregnancy Physical Activity Questionnaire (PPAQ), which was translated and cross-culturally adapted for the Brazilian population, according to Silva et al. [38]. In this PPAQ, the type of exercise, intensity, duration and frequency was recorded per day during the current gestational trimester (i.e., based on activities performed in the last three months). To compute the average daily energy expenditure, we follow the instructions described by Silva et al. [38] and thus the pregnant women were classified according to their intensity level of daily physical activity as sedentary, light or moderate.

### 2.5. Exposure Variable

Pre-pregnancy weight (kg) was self-reported or extracted from the pregnant woman’s medical record, referring to the measurement performed in the first trimester. Height (m) was measured in duplicate to the nearest 0.1 cm using a portable stadiometer (Seca Ltd., Hamburg, Germany) attached to the wall, with a limit of 220 cm. The mean between the two measures was calculated. Pre-pregnancy BMI (weight (kg)/height (m^2^)) was subsequently calculated and women were classified as having excess weight (yes/no) according to the World Health Organisation BMI cut-off point for excess weight (≥25 kg/m^2^). In Brazil, self-reported pre-pregnancy weight can be used to calculate BMI when an early measurement of weight during pregnancy is not available [39].

### 2.6. Outcome Variables

The outcomes were changes in CRP (mg/L), IL-6 (pg/mL), IL-10 (ng/mL) and MMP-9 (ng/mL) serum concentrations over pregnancy and early postpartum. CRP levels (serum) were measured by the immunoturbidimetric method, MMP-9 (serum), IL-6, and IL-10 levels (plasma) were measured by xMap methodology in the Luminex 200 equipment (software xPonent/Analyst version 4.2, Millipore, St Charles, MO, USA).

### 2.7. Data Analysis

The sample characteristics were described using medians, interquartile ranges (IQR), absolute values (*n*) and relative frequencies (%). The ANOVA test was used to determine statistical significance between continuous variables and the Chi-square test for categorical variables.

Bivariate longitudinal linear regressions were performed to investigate the associations of maternal, sociodemographic, biochemical, and nutritional factors with the inflammatory biomarkers’ level during pregnancy and postpartum (Supplementary material Table S1). An alpha of 0.2 was used in bivariate pre-screening of candidate variables to ensure that all potential explanatory variables were considered. After this exploratory bivariate analysis, we examined the correlation between variables and selected the co-variates to be considered in the multivariate model. Pocket depth and CAL were strongly correlated. Therefore, these two variables were not simultaneously included in the multivariate models.

Multivariate longitudinal generalised linear mixed-effects (LME) were performed to assess the influence of excess pre-pregnancy BMI (yes/no) on the levels of inflammatory markers throughout pregnancy and postpartum (reported coefficients (β) and their 95% confidence interval (CI)). We explored some transformations (rank, log-rank, etc.) to deal with data skewness. However, data transformations did not improve the model fit significantly. Therefore, the results were presented in the original unit of analysis, but outliers were removed. The gestational age (in weeks) and the women’s identification (ID) were included in all LME models as random effects ID to adjust for the overall and the individual variations in the outcomes over time. This means that we modelled the average effect for each subject as a random factor. The periodontal treatment allocation and the milk fortification are both directly linked to the study outcomes (changes in inflammatory markers) and were therefore assessed as possible random effects and included in the final model regardless of the *p*-value. This ensure that any observed association between pre-pregnancy BMI and inflammatory markers was independent of any potential intervention effect. CAL was also included in the adjusted models. All other variables were considered as fixed effects only and were retained in the model based on a significance level of 0.05.

The intervention groups were balanced at baseline, demonstrating that the randomisation strategy was effective (data not shown). Further details can be found elsewhere [40]. Randomisation was stratified according to the baseline smoking habits (yes/no). Therefore, this variable was not considered as a covariate in the regression models. The only exposure variables measured over pregnancy and postpartum were the number of acute infection episodes (urinary, throat, flu, and other) and the use of anti-inflammatory drugs. All other variables were measured at baseline, only.

Statistical analyses were performed using R version 3.6.1 (R Core Team, Vienna, Austria) [41] with the “lme4” package to perform LME analyses [42].

### 2.8. Ethics

This study was conducted according to the guidelines of the Declaration of Helsinki. All participants signed informed consent for participation in the study. The IMPROVE trial protocol was submitted and approved by the Ethics Committee of School Maternity of the Federal University of Rio de Janeiro (Brazil), on 27 April 2016 (nr. 1.516.656). The trial was registered in the ClinicalTrials.gov database (NCT, NCT03148483. Registered on 11 May 2017, https://clinicaltrials.gov/ct2/show/NCT03148483 (accessed on 18 January 2019), and the study protocol was published [24].

## 3. Results

The 68 participants’ median age was 28.5 (IQR = 7.0) years, and gestational age at baseline was 16.4 (4.1) weeks. The median years of education were 12.0 (3.0) years, and the monthly per capita income was USD 130.0 (104.1). Eighty-seven per cent (87%) of pregnant women lived with their partners, 64.7% had at least one previous delivery and 88.2% declared themselves racially mixed or black. The median value for BMI was 26.3 (9.5) kg/m^2^. The median values for pocket depth, CAL and the frequency of BOP were 4.2 (0.3) mm, 4.2 (0.3) mm, and 16.0% (21.0%), respectively at baseline. Women with pre-pregnancy excess weight were older and had higher median values of fasting insulin and glucose (*p* < 0.001) compared to women without an excess of weight at baseline (Table 1).

Periodontal parameters tended to improve (negative delta values) in women with no excess weight compared to those with excess weight; however, this improvement was not statistically significant (*p* > 0.05). Overall, periodontal parameters did not change significantly between the second trimester of pregnancy and early postpartum (Table 2).

The median CRP levels ranged from 8.6 (9.9) mg/L at baseline to 4.5 (5.9) mg/L in the postpartum (*p* < 0.001). IL-6 concentrations varied from 1.2 (0.6) (pg/mL) at baseline to 1.3 (0.6) (pg/mL) in the postpartum period (*p* = 0.012). IL-10 levels did not change significantly throughout the study period: 2.9 (2.0) (pg/mL) at baseline and 3.2 (1.9) (pg/mL) in postpartum (*p* = 0.074). MMP-9 levels varied from 4.5 (1.6) to 3.0 (2.2) ng/mL in the postpartum (*p* < 0.001) (data not shown).

In the bivariate analysis, pre-pregnancy excess weight was associated with CRP levels at baseline (*p* < 0.01) (Supplementary material Table S1). However, there were no significant associations between pre-pregnancy excess weight and IL-6, IL-10 and MMP-9 in the bivariate analyses.

Longitudinal adjusted models showed that pre-pregnancy excess weight (β = 4.39; 95% CI, 2.12–6.65) was associated with increased CRP levels from pregnancy to postpartum (Table 3). Pre-pregnancy BMI was not associated with longitudinal changes in IL-6, IL-10 and MMP-9 concentration in the multivariable analysis (data not shown).

## 4. Discussion

Pre-pregnancy BMI was directly associated with changes in CRP levels, suggesting that women with a higher BMI early in pregnancy increased their CRP levels from pregnancy to postpartum more so than women with a healthy weight. There were no significant associations with changes in IL-6, MMP-9 and IL-10 concentrations over time. To the best of our knowledge, no previous studies have examined the changes of several inflammatory biomarkers during pregnancy and postpartum in women with periodontitis from a low socioeconomic setting. Thus, a direct comparison between our results and findings from other studies is limited.

It is known that pre-pregnancy obesity and several lifestyle factors, including a diet rich in meat and processed foods, correlate directly with circulating concentrations of inflammatory biomarkers, such as CRP and IL-6 [22,23]. Oliveira et al. [21] studied 115 pregnant women from Brazil and observed that serum CRP concentrations progressively increased throughout pregnancy and that pre-pregnancy BMI was directly associated with subsequent increases in CRP concentrations. Moreso, McDade et al. [43] conducted a study with 309 young pregnant women and observed that pre-pregnancy BMI was directly associated with CRP in the third gestational trimester. In pregnant women, there is an overlap of metabolic and inflammatory pathways [44,45], and a possible explanation for these findings is that macrophages, that are related to the inflammatory response [43,45] and adipocytes that under normal conditions store lipids and regulate metabolic homeostasis [43], have common embryonic origins and might be able, in particular situations as maternal excess of weight, to produce the same components, including inflammatory markers. However, little is known about the relationship between BMI and the dynamics of the concentrations of other inflammatory biomarkers, such as interleukins and MMP-9 in the context of pregnancy, and even less is known about these relationships in pregnant women with periodontitis.

Although markers of systemic inflammation such as CRP raise slightly at the beginning of gestation, indicating appropriate placentation [46], a persistent chronic systemic inflammatory response with increased cytokines and CRP levels is associated with placental dysfunction and adverse gestational outcomes [47], including preeclampsia [48], ruptured membranes complicated by chorioamnionitis [49], and preterm labour [50]. There is evidence supporting that levels of CRP increase during pregnancy above non-pregnant levels and then gradually reduce after childbirth [46]. However, the causes of CRP changes throughout pregnancy are still not fully known [46]. High levels of CRP in the first trimester have been reported [51], and more recently, it was shown that pregnant women with elevated CRP levels at 9 (±13) weeks were more likely to develop gestational diabetes mellitus [52] and preeclampsia [53] than pregnant women with lower levels. We found that excess pre-pregnancy weight is associated with a 4.39 mg/L increase in CRP compared to normal pre-pregnancy weight. However, the clinical relevance of this finding is not clear. Nakishbandy & Barawi [54], found that pregnant women with CRP levels above 1 mg/L were at high risk of preterm delivery, while Dodd’s & Iams [55] observed that the risk of preterm delivery increased among women with a CRP level of 8 mg/L or greater.

The potential intervention effect of this feasibility trial on periodontal measurements and other parameters was fully reported in our previous paper [40]. In brief, we found that mean BOP was significantly reduced in the early periodontal treatment arm. Concomitantly, this clinical parameter worsened in the late periodontal treatment arm. No significant differences were observed regarding pocket depth and CAL. Given the above and evidence from other literature that periodontal treatment can influence inflammatory markers [56], we explored the associations between the three periodontal parameters (pocket depth, CAL and BOP) and inflammatory markers in the bivariate analysis (Supplemental Table S1). All three parameters were directly and significantly associated with changes in CPR from baseline (*p* < 0.001) in the bivariate analyses but did not remain significant in the adjusted models. CAL was forced in adjusted models, but it did not influence the direction or strength of the associations.

To the best of our knowledge, we did not include any women with aggressive periodontitis. This is because women with aggressive periodontitis were more likely to have poor overall oral health (e.g., presence of extensive caries) and therefore was not eligible to be included in the study. Additionally, our study population was relatively young and most of the eligible women presented with mild chronic periodontitis. Recruiting individuals with severe chronic periodontitis only, which tends to develop with age, would not be feasible [28]. Therefore, periodontitis was defined as the presence of one or more teeth with at least one periodontal site with ≥4 mm of CAL and with the presence of BOP on the same site. The presence of BOP ensured the existence of local inflammation.

It is important to emphasise the relationship between periodontitis and inflammatory markers in the context of obesity. There is compelling evidence pointing out the increased risk of periodontitis in individuals with excessive weight [57]. Although the underlying mechanism remains unclear, it is suggested that it might be mediated by insulin resistance due to chronic inflammatory and oxidative stress [57]. However, currently, the available evidence on the success of periodontal therapy on clinical periodontal parameters and levels of pro-inflammatory cytokines in obese individuals remains inconclusive [58,59]. Despite the limited evidence on the effectiveness of periodontal therapy on patients with obesity, oral healthcare professionals need to be aware of the intricate relationship between obesity and periodontitis, especially in pregnant and early postpartum women and need to inform them about the importance of maintaining a healthy body weight and having good oral hygiene practice [59].

Our findings should be interpreted with caution as it was an exploratory secondary analysis of a feasibility clinical trial. Therefore, the study was limited by the small sample size and the loss of follow-up. In total, 68 women were evaluated, and 15 (21.7%) did not complete the follow-up. However, no differences were observed in baseline characteristics for the women who completed and those who dropped out of the study [28], suggesting a low probability of selective losses. The use of self-reported pre-pregnancy BMI may be a limitation of our study. However, Carrilho et al. [39] observed that self-reported pre-pregnancy weight could be used in a Brazilian population to calculate BMI when an early measurement of weight during pregnancy is not available. The generalisability of our findings might be limited. Our results could be potentially generalised to other South American populations living in a similar environment.

The main strength of this study was the repeated biomarker measures from pregnancy up to postpartum and the use of longitudinal modelling. The model is robust for correlations between repeated measurements and incomplete follow-up data on the outcome variable by considering the common effects of participants in the same group-fixed effects and specific effects of each participant-random effect [60]. This procedure improved the accuracy of estimates and required fewer observations, which is advantageous for studies with small sample sizes. Another strength was the similarity in periodontal status across group allocation [40].

Some research and clinical implications of our findings should be highlighted. A higher pre-pregnancy BMI was associated with increased levels of inflammatory biomarkers throughout pregnancy and postpartum in women with periodontitis. This chronic inflammatory profile may lead to adverse pregnancy outcomes [3,18,20]. In this scenario, maternal excess weight and inflammation are risk factors for adverse pregnancy and birth outcomes. However, both periodontitis and excess weight are preventable risk factors, and they should be targeted for early interventions. Finally, it is important to mention that oral health screening is not offered to all pregnant women in developing and some developed countries, and oral health, in general, is often neglected [61]. Furthermore, healthcare professionals should be aware of the health consequences for both mother and offspring related to periodontitis and secure strategies to minimise the negative health impacts, particularly among those with other risk factors, including those with excess weight.

## 5. Conclusions

Pre-pregnancy BMI was significantly associated with increased CRP levels from pregnancy to the early postpartum period among pregnant Brazilian women with periodontitis. More research to investigate if predictors of changes in inflammatory markers can be used as prognostic factors for gestational outcomes.

## Figures and Tables

**Table 1:** Baseline ^1^ characteristics of pregnant women with periodontitis according to pre-pregnancy BMI categories.

Maternal Characteristics	Total ^2^	Pre-Pregnancy BMI Categories		*p* *
		No Excess Weight (≤24.9 kg/m^2^)	Excess Weight (≥25 kg/m^2^)	
Age (years)	28.5 (7.0)	25.0 (8.0)	30.0 (8.0)	0.009
Gestational age (weeks)	16.4 (4.1)	17.0 (4.6)	16.4 (3.7)	0.896
Years of education (years)	12.0 (3.0)	12.0 (3.0)	12.0 (3.0)	0.926
Monthly per capita income (US$) ^3^	130.0 (104.1)	120.0 (78.3)	151.1 (152.3)	0.185
Pre-pregnancy BMI (kg/m^2^)	26.3 (9.5)	21.4 (2.2)	30.5 (6.2)	<0.001
Gestational weight gain (kg)	10.1 (8.7)	11.0 (6.1)	6.9 (12.7)	0.033
Insulin (µU/mL)	7.6 (5.2)	4.9 (3.8)	9.6 (4.9)	<0.001
Fasting glucose (mg/dL)	72.5 (9.5)	68.0 (7.0)	75.0 (8.0)	0.001
Energy intake (kcal/d)	4054 (2334)	4216 (2549)	3927 (2458)	0.278
Calcium intake (mg/d)	1037 (636)	953 (478)	1045 (944)	0.867
Vitamin D intake (mcg/d)	5.3 (4.4)	5.6 (4.2)	5.0 (3.2)	0.995
Inflammatory diet index ^4^	0.8 (2.1)	1.1 (2.0)	0.8 (1.9)	0.724
Pocket depth (mm)	4.2 (0.3)	4.2 (0.2)	4.3 (0.2)	0.406
CAL (mm)	4.2 (0.3)	4.2 (0.3)	4.3 (0.3)	0.360
BOP (%)	16.0 (21.0)	15.0 (21.0)	16.0 (22.0)	0.823
Living with a partner				
Yes	59 (86.8)	24 (82.8)	35 (89.7)	0.401
No ^5^	9 (13.2)	5 (17.2)	4 (10.3)	
Parity (number of parturitions)				
0	24 (35.3)	12 (41.4)	12 (30.8)	0.344
≥1	44 (64.7)	17 (58.6)	27 (69.2)	
Self-reported skin colour				
White	8 (11.8)	4 (13.8)	4 (10.3)	0.654
Mixed or black	60 (88.2)	25 (86.2)	35 (89.7)	
Current smoking				
No	60 (88.2)	27 (93.1)	33 (84.6)	0.283
Yes	8 (11.8)	2 (6.9)	6 (15.4)	
Alcohol intake				
No	57 (83.8)	25 (86.2)	32 (82.0)	0.645
Yes	11 (16.2)	4 (13.8)	7 (18.0)	
Physical Activity				
Sedentary and light	42 (61.8)	20 (69.0)	22 (56.4)	0.292
Moderate	26 (38.2)	9 (31.0)	17 (43.6)	
Sun exposure				
<30 min/day	48 (81.4)	18 (81.8)	30 (81.1)	0.944
>30 min/day	11 (18.6)	4 (18.2)	7 (18.9)	

^1^ Baseline was between gestational weeks 11 and 21. ^2^ *n* = 68. ^3^ Value was originally measured in Brazilian Reais (BRL) but converted to USA dollars (USD). Exchange rate in February 2019, BRL 3.75 = USD 1. ^4^ Score classifies diets on a continuum from maximally anti-inflammatory (negative values) to maximally pro-inflammatory (positive values). ^5^ Other = Not living with a partner or do not have a partner. BMI, Body Mass Index. * *p*-value refers to the Kruskal–Wallis test between continuous exposure variables and BMI categories and refers to Chi-square test between categorical exposure variables and BMI categories.

**Table 2:** Change in periodontal health measures (delta) ^1^ among pregnant women with periodontitis according to pre-pregnancy BMI categories.

Periodontal Parameters	Total ^2^	Pre-Pregnancy BMI Categories		*p*
		No Excess Weight (≤24.9kg/m^2^)	Excess Weight (≥25 kg/m^2^)	
	Median (IQR)			
Δ Pocket depth (mm) ^1^	0.0 (0.3)	−0.1 (0.2)	0.0 (0.3)	0.171
Δ CAL (mm) ^1^	0.0 (0.3)	−0.1 (0.2)	0.0 (0.3)	0.139
Δ BOP (%) ^1^	0.0 (0.2)	−0.1 (0.2)	0.0 (0.2)	0.080

^1^ The difference between T2 and T0. ^2^ *n* = 68.

**Table 3:** Adjusted longitudinal linear regression model for excess weight (yes/no) based on pre-pregnancy BMI and CRP levels from pregnancy to postpartum among pregnant women with periodontitis.

Variables			
CRP Change	β	95% CI	*p*-Value
Pre-pregnancy excess of weight (no/yes) ^1^	4.39	2.12–6.65	<0.01
Insulin (µU/mL)	0.35	0.11–0.58	<0.01
CAL (mm)	3.08	−1.93–8.08	0.23
**Restricted Maximum Likelihood Estimation (REML) at the convergence**	1095.4		
**Variance components**	**Variance**	**SD**	95% CI
Woman identifier (ID)	15.37	3.92	0–7.89
Gestational age (wk)	0.003	0.06	0–0.18
Residual	37.36	6.11	5.33–7.00
**Lilliefors (Kolmogorov-Smirnov) normality test**	**D**		*p*-Value
for residuals of fixed effects	0.12		*<0.01*
for random effects	0.31		*<0.01*

β is the angular coefficient. ^1^ Assessed by pre-pregnancy BMI (Kg/m^2^). Excess of weight = overweight + obesity. Categorical variables for which the first category is the reference. Italic: normal distribution an alpha 0.01. CRP: C-reactive protein; wk: weeks; SD: Standard Deviation; D: Statistical distribution of normality. Variables were retained in the final model if *p*-value was <0.05.

## Data Availability

The datasets used or analysed during the current study are available from the corresponding author on reasonable request.

## Supplementary Materials

The following supporting information can be downloaded at: https://www.mdpi.com/article/10.3390/ijerph19052705/s1, Table S1: Longitudinal bivariate linear regression model for CRP, IL-6 and IL-10 interleukins, and MMP-9 levels in pregnant women with periodontitis in Rio de Janeiro, Brazil.

## Abbreviations

BOP: bleeding on probing; CAL: clinical attachment loss; CRP: C-reactive protein; BMI: Body Mass Index; DII: Dietary Inflammatory Index; FHS: family health strategy; IL-6: Interleukin-6; IL-10: Interleukin-10; IQR: interquartile range; WK: week; MMP-9: Matrix metallopeptidase 9; PT: periodontal therapy; SD: standard deviation.

## References

[1] Chrisopoulos S., Harford J.E., Ellershaw A. (2016). Oral Health and Dental Care in Australia: Key Facts and Figures 2015.

[2] Figuero E., Carrillo-de-Albornoz A., Martín C., Tobías A., Herrera D. (2013). Effect of pregnancy on gingival inflammation in systemically healthy women: A systematic review. J. Clin. Periodontol..

[3] Ide M., Papapanou P.N. (2013). Epidemiology of association between maternal periodontal disease and adverse pregnancy outcomes—Systematic review. J. Clin. Periodontol..

[4] Boggess K.A., Lieff S., Murtha A.P., Moss K., Beck J., Offenbacher S. (2003). Maternal periodontal disease is associated with an increased risk for preeclampsia. Obstet. Gynecol..

[5] Xiong X., Buekens P., Vastardis S., Yu S.M. (2007). Periodontal disease and pregnancy outcomes: State-of-the-science. Obstet. Gynecol. Surv..

[6] Swamy G., Murtha A., Jared H., Boggess K., Lieff S., Heine P. (2002). Post-cesarean infection in women with periodontal disease. Am. J. Obstet. Gynecol..

[7] Nibali L., D’Aiuto F., Griffiths G., Patel K., Suvan J., Tonetti M.S. (2007). Severe periodontitis is associated with systemic inflammation and a dysmetabolic status: A case-control study. J. Clin. Periodontol..

[8] Duarte P.M., da Rocha M., Sampaio E., Mestnik M.J., Feres M., Figueiredo L.C., Bastos M.F., Faveri M. (2010). Serum levels of cytokines in subjects with generalized chronic and aggressive periodontitis before and after non-surgical periodontal therapy: A pilot study. J. Periodontol..

[9] Van Dyke T.E., Van Winkelhoff A.J. (2013). Infection and inflammatory mechanisms. J. Clin. Periodontol..

[10] Ardila C.M., Lafaurie G.I. (2010). Asociación entre *porphyromona gingivalis* y proteína C reactiva en enfermedades sistêmicas inflamatorias. Av. Periodoncia.

[11] Jonakait G.M. (2007). The effects of maternal inflammation on neuronal development: Possible mechanisms. Int. J. Dev. Neurosci..

[12] Marnell L., Mold C., Du Clos T.W. (2005). C-reactive protein: Ligands, receptors and role in inflammation. Clin. Immunol..

[13] Escobar-Arregoces F., Latorre-Uriza C., Velosa-Porras J., Roa-Molina N., Ruiz A.J., Silva J., Arias E., Echeverri J. (2018). Inflammatory response in pregnant women with high risk of preterm delivery and its relationship with periodontal disease: A pilot study. Acta Odontol. Latinoam..

[14] Lian Y.Y., He H.H., Zhang C.Z., Li X.C., Chen Y.H. (2019). Functional characterization of a matrix metalloproteinase 2 gene in Litopenaeus vannamei. Fish Shellfish. Immunol..

[15] Yu A.P., Tam B.T., Yau W.Y., Chan K.S., Yu S.S., Chung T.L., Siu P.M. (2015). Association of endothelin-1 and matrix metallopeptidase-9 with metabolic syndrome in middle-aged and older adults. Diabetol. Metab. Syndr..

[16] Orange S., Rasko J.E., Thompson J.F., Vaughan J., Olive E., Pedler M., Horvath J.S., Hennessy A. (2005). Interleukin-10 regulates arterial pressure in early primate pregnancy. Cytokine.

[17] Kalkunte S., Nevers T., Norris W.E., Sharma S. (2011). Vascular IL-10: A protective role in preeclampsia. J. Reprod. Immunol..

[18] Mesa F., Pozo E., O’Valle F., Puertas A., Magan-Fernandez A., Rosel E., Bravo M. (2016). Relationship between periodontal parameters and plasma cytokine profiles in pregnant woman with preterm birth or low birth weight. Clin. Oral Investig..

[19] Mahapatra A., Nayak R., Satpathy A., Pati B.K., Mohanty R., Mohanty G., Beura R. (2020). Maternal periodontal status, oral inflammatory load, and systemic inflammation are associated with low infant birth weight. J. Periodontol..

[20] Oliveira L.C., Franco-Sena A.B., Farias D.R., Rebelo F., Kac G. (2017). Maternal CRP concentrations during pregnancy and birth weight in a prospective cohort in Rio de Janeiro, Brazil. J. Matern. Fetal Neonatal Med..

[21] Oliveira L.C., Franco-Sena A.B., Rebelo F., Farias D.R., Lepsch J., Lima N.S., Kac G. (2015). Factors associated with maternal serum CRP throughout pregnancy: A longitudinal study in women of Rio de Janeiro, Brazil. Nutrition.

[22] Barbaresko J., Koch M., Schulze M.B., Nothlings U. (2013). Dietary pattern analysis and biomarkers of low-grade inflammation: A systematic literature review. Nutr. Rev..

[23] Calder P.C., Ahluwalia N., Brouns F., Buetler T., Clement K., Cunningham K., Esposito K., Jönsson L.S., Kolb H., Lansink M. (2011). Dietary factors and low-grade inflammation in relation to overweight and obesity. Br. J. Nutr..

[24] Cocate P.G., Kac G., Heitmann B.L., Nadanovsky P., da Veiga M.C., Benaim C., Schlüssel M.M., de Castro M.B.T., Alves-Santos N.H., Baptista A.F. (2019). Calcium and vitamin D supplementation and/or periodontal therapy in the treatment of periodontitis among Brazilian pregnant women: Protocol of a feasibility randomized controlled trial (the IMPROVE trial). Pilot Feasibility Stud..

[25] IBGE (Instituto Brasileiro de Geografia e Estatística) (2017). Estimativas da População Residente Com Data de Referência 1º de Julho De 2017.

[26] DESANS (Departamento Geral de Segurança Alimentar e Nutricional Sustentável) (2012). Diagnóstico Situacional do Município de Duque de Caxias.

[27] Palmeira P.A., Bem-Lignani J., Maresi V.A., Mattos R.A., Interlenghi G.S., Salles-Costa R. (2019). Temporal Changes in the Association Between Food Insecurity and Socioeconomic Status in Two Population-Based Surveys in Rio de Janeiro, Brazil. Soc. Indic. Res..

[28] Adegboye A., Santana D., Cocate P.G., Benaim C., Santos P., Heitmann B., Carvalho M.C.D.V.S., Schlüssel M.M., De Castro M.B.T., Kac G. (2020). Vitamin D and Calcium Milk Fortification in Pregnant Women with Periodontitis: A Feasibility Trial. Int. J. Environ. Res. Public Health.

[29] Yeung C.A. (2014). Book review: Oral health surveys: Basic methods, 5th ed. Br. Dent. J..

[30] Hutcheon J.A., Bodnar L.M., Joseph K.S., Abrams B., Simhan H.N., Platt R.W. (2012). The bias in current measures of gestational weight gain. Paediatr. Perinat. Epidemiol..

[31] Barbieri P., Nishimura R., Crivellenti L., Sartorelli D. (2013). Relative validation of a quantitative FFQ for use in Brazilian pregnant women. Public Health Nutr..

[32] Vilela A.A., Farias D.R., Eshriqui I., Vaz J.S., Franco-Sena A.B., Castro M.B., Olinto M.T.A., Machado S.P., Da Silva A.A.M., Kac G. (2014). Prepregnancy healthy dietary pattern is inversely associated with depressive symptoms among pregnant Brazilian women. J. Nutr..

[33] NEPA (Núcleo de Estudos e Pesquisas em Alimentação) (2011). Tabela Brasileira de Composição de Alimentos (TACO).

[34] IBGE (Instituto Brasileiro de Geografia e Estatística) (2011). Pesquisa de Orçamentos Familiares. Tabela de Composição Nutricional dos Alimentos Consumidos no Brasil.

[35] USDA (United States Department of Agriculture) (2011). USDA National Nutrient Database for Standard Reference.

[36] Shivappa N., Steck S.E., Hurley T.G., Hussey J.R., Hébert J.R. (2014). Designing and developing a literature-derived, population-based dietary inflammatory index. Public Health Nutr..

[37] Cavicchia P.P., Steck S.E., Hurley T.G., Hussey J.R., Ma Y., Ockene I.S., Hébert J.R. (2009). A new dietary inflammatory index predicts interval changes in high-sensitivity c-reactive protein. J. Nutr..

[38] Silva F.T., Júnior E.A., Santana E.F.M., Lima J.W.O., Cecchino G.N., Da Silva Costa F. (2015). Translation and cross-cultural adaptation of the Pregnancy Physical Activity Questionnaire (PPAQ) to the Brazilian population. Čes. Gynek..

[39] Carrilho T., Rangel T., Rasmussen K., Farias D.R., Costa N., Batalha M., Reichenheim M., Ohuma E.O., Hutcheon J.A., Kac G. (2020). Agreement between self-reported pre-pregnancy weight and measured first-trimester weight in Brazilian women. BMC Pregnancy Childbirth.

[40] Adegboye A., Santana D., Santos P., Cocate P.G., Benaim C., Castro M.B., Schlüssel M.M., Kac G., Heitmann B.L. (2021). Exploratory Efficacy of Calcium-Vitamin D Milk Fortification and Periodontal Therapy on Maternal Oral Health and Metabolic and Inflammatory Profile. Nutrients.

[41] R Core Team (2014). R: A Language and Environment for Statistical Computing.

[42] Bates D., Maechler M., Bolker B., Walker S. (2015). Fitting Linear Mixed-Effects Models Using lme4. J. Stat. Softw..

[43] McDade T.W., Borja J.B., Largado F., Adair L.S., Kuzawa C.W. (2016). Adiposity and Chronic Inflammation in Young Women Predict Inflammation during Normal Pregnancy in the Philippines. J. Nutr..

[44] Lorgeril M., Salen P., Martin J.L., Monjaud I., Delaye J., Mamelle N. (1999). Mediterranean diet, traditional risk factors, and the rate of cardiovascular complications after myocardial infarction: Final report of the Lyon Diet Heart Study. Circulation.

[45] Uriza C.L., Velosa-Porras J., Roa N.S., Lara S.M.Q., Silva J., Ruiz A.J., Arregoces F.M.E. (2018). Periodontal Disease, Inflammatory Cytokines, and PGE2 in Pregnant Patients at Risk of Preterm Delivery: A Pilot Study. Infect. Dis. Obstet. Gynecol..

[46] Sacks G., Seyani L., Lavery S., Trew G. (2004). Maternal C-reactive protein levels are raised at 4 weeks gestation. Hum. Reprod..

[47] Witteveen A.B., Henrichs J., Bellers M., van Oenen E., Verhoeven C.J., Vrijkotte T. (2020). Mediating role of C-reactive protein in associations between pre-pregnancy BMI and adverse maternal and neonatal outcomes: The ABCD-study cohort. J. Matern. Fetal Neonatal Med..

[48] Teran E., Escudero C., Moya W., Flores M., Vallance P., Lopez-Jaramillo P. (2001). Elevated C-reactive protein and pro-inflammatory cytokines in Andean women with pre-eclampsia. Int. J. Gynaecol. Obstet..

[49] Yoon B.H., Jun J.K., Park K.H., Syn H.C., Gomez R., Romero R. (1996). Serum C-reactive protein, white blood cell count, and amniotic fluid white blood cell count in women with preterm premature rupture of membranes. Obstet. Gynecol..

[50] Hvilsom G.B., Thorsen P., Jeune B., Bakketeig L.S. (2002). C-reactive protein: A serological marker for preterm delivery?. Acta. Obstet. Gynecol. Scand..

[51] Rebelo I., Carvalho-Guerra F., Pereira-Leite L., Quintanilha A. (1995). Lactoferrin as a sensitive blood marker of neutrophil activation in normal pregnancies. Eur. J. Obstet. Gynecol. Reprod. Biol..

[52] Wolf M., Sandler L., Hsu K., Vossen-Smirnakis K., Ecker J.L., Thadhani R. (2003). First trimester C-reactive protein and subsequent gestational diabetes. Diabetes Care.

[53] Wolf M., Kettyle E., Sandler L., Ecker J.L., Roberts J., Thadhani R. (2001). Obesity and preeclampsia: The potential role of inflammation. Obstet. Gynecol..

[54] Nakishbandy B.M., Barawi S.A.M. (2014). Level of C-reactive protein as an indicator for prognosis of premature uterine contractions. J. Prenat. Med..

[55] Dodds W.G., Lams J.D. (2003). Maternal C-reactive protein and preterm labor. J. Repord. Med..

[56] D’Aiuto F., Gkranias N., Bhowruth D., Khan T., Orlandi M., Suvan J., Masi S., Tsakos G., Hurel S., Hingorani A. (2018). Systemic effects of periodontitis treatment in patients with type 2 diabetes: A 12 month, single-centre, investigator-masked, randomised trial. Lancet Diabetes Endocrinol..

[57] Martinez-Herrera M., Silvestre-Rangil J., Silvestre F.J. (2017). Association between obesity and periodontal disease. A systematic review of epidemiological studies and controlled clinical trials. Med. Oral Patol. Oral Cir. Bucal..

[58] Arboleda S., Vargas M., Losada S., Pinto A. (2019). Review of obesity and periodontitis: An epidemiological view. Br. Dent. J..

[59] Akram Z., Safii S.H., Vaithilingam R.D., Baharuddin N.A., Javed F., Vohra F. (2016). Efficacy of non-surgical periodontal therapy in the management of chronic periodontitis among obese and non-obese patients: A systematic review and meta-analysis. Clin. Oral Investig..

[60] Spyrides M.A.C., Struchiner C.J., Barbosa M.T., Kac G., Kac G., Sichieri R., Gigante D.P. (2007). Data Analysis with Repeated Measures. Nutritional Epidemiology.

[61] Peres M.A., Macpherson L., Weyant R.J., Daly B., Venturelli R., Mathur M.R., Listl S., Celeste R.K., Guarnizo-Herreño C.C., Kearns C. (2019). Oral diseases: A global public health challenge. Lancet.

