# Supplementary material for: Association between Pre-Pregnancy BMI and Inflammatory Profile Trajectories during Pregnancy and Postpartum in Brazilian Women with Periodontitis: The IMPROVE Trial
Source: Int J Environ Res Public Health. 2022 Feb 25;19(5):2705. doi: 10.3390/ijerph19052705 (PMC8909899; doi:10.3390/ijerph19052705)
Supplement: Supplementary file 1 [file ijerph-19-02705-s001.zip › ijerph-1536029-supplementary.pdf]

Supplemental Table S1. Longitudinal bivariate linear regression model for CRP, IL-6 and IL-10 interleukins, and MMP-9 levels in pregnant women with periodontitis in Rio de Janeiro, Brazil.

**Variables**	**CRP**			**IL-6**			**IL-10**			**MMP-9**		
	β[1]	**95% CI**	***p* value**	β[1]	**95% CI**	***p* value**	β[1]	**95% CI**	***p* value**	β[1]	**95% CI**	***p* value**
Age (y)	0.165	-0.005/0.336	**0.059**	-0.045	-0.093/0.001	**0.061**	-0.078	-0.141/-0.013	**0.016**	0.042	0.002/0.081	**0.036**
Years of education (y)	-0.288	-0.742/0.162	0.212	0.096	-0.028/0.220	**0.131**	0.010	-0.158/0.180	0.902	0.116	0.010/0.220	**0.029**
Monthly per-capita income (US$)	0.0007	-0.0015/0.003	0.522	-0.0003	-0.0009/0.0003	0.338	-0.0004	-0.0012/0.0004	0.368	0.0005	0.00001/0.001	**0.044**
Marital status (other/living with partner)[2]	0.330	-2.915/3.602	0.842	-1.000	-1.877/-0.135	**0.024**	0.034	-1.158/1.255	0.955	0.582	-0.150/1.320	**0.122**
Parity (number of parturitions)	-0.038	-0.908/0.8333	0.932	-0.286	-0.526/0.046	**0.020**	-0.388	-0.708/-0.061	**0.018**	-0.132	-0.332/0.067	**0.195**
Alcohol intake (no/yes)[2]	-0.239	-2.960/2.479	0.863	-0.530	-1.280/0.220	**0.168**	-0.458	-1.476/0.560	0.378	-0.320	-0.948/0.318	0.320
Prepregnancy BMI (kg/m^2)	0.320	0.184/0.457	**<0.001**	0.059	0.020/0.097	**0.002**	0.002	-0.051/0.061	0.913	0.001	-0.032/0.034	0.952
Gestational weight gain (kg)	-0.060	-0.203/0.083	0.413	-0.027	-0.076/0.023	0.289	0.013	-0.040/0.065	0.633	-0.004	-0.036/0.030	0.827
Insulin (μU/mL)	0.500	0.268/0.739	**<0.001**	0.034	-0.023/0.092	0.247	0.040	-0.048/0.132	0.365	0.096	0.042/0.151	**<0.001**
Energy intake (kcal/d)	0.0001	-0.0003/0.0005	0.629	-0.0001	-0.0001/0.00002	**0.141**	-0.0001	-0.0002/0.0001	0.316	-0.00006	-0.0001/0.00002	**0.151**
Calcium intake (mg/d)[3]	0.001	-0.0002/0.002	**0.105**	-0.0003	-0.0007/0.00007	**0.112**	-0.0002	-0.0007/0.0002	0.398	-0.0001	-0.0004/0.0002	0.470
Inflammatory diet index	-0.252	-0.763/0.258	0.334	0.144	0.003/0.285	**0.046**	-0.015	-0.209/0.176	0.872	0.113	-0.005/0.230	**0.060**
Number of infections	-0.127	-0.368/0.111	0.299	0.026	-0.039/0.0922	0.433	0.149	0.062/0.236	**<0.001**	-0.002	-0.058/0.053	0.924
Anti-inflammatory use (no/yes)[2]	1.230	-0.895/3.363	0.258	0.025	-0.559/0.610	0.931	-0.253	-1.046/0.553	0.533	0.498	0.006/0.990	**0.048**
Periodontal treatment (no/yes)[2]	-1.00	-2.980/0.978	0.322	0.958	0.424/1.492	**<0.001**	0.717	-0.023/1.451	**0.057**	-0.295	-0.752/0.160	0.206
Vit D/ milk fortification (no/yes)[2]	2.054	0.091/3.997	**0.040**	-0.134	-0.682/0.414	0.632	0.413	-0.343/1.150	0.273	0.192	-0.264/0.650	0.410
Pocket depth (mm)	6.539	3.932/9.127	**<0.001**	0.538	-0.244/1.320	**0.177**	1.361	0.404/2.318	**0.005**	-0.232	-0.773/0.306	0.398
CAL (mm)	6.198	3.828/8.563	**<0.001**	0.152	-0.646/0.950	0.709	0.313	-0.603/1.234	0.503	-0.529	-1.069/0.009	**0.054**
BOP (%)	8.675	4.771/12.552	**<0.001**	2.008	0.863/3.150	**<0.001**	1.380	-0.034/2.796	**0.056**	-0.268	-1.063/0.526	0.508
Δ Pocket depth (mm)[4]	-4.729	-7.292/-2.156	**<0.001**	-0.487	-1.318/0.342	0.251	-2.793	-3.783/-1.802	**<0.001**	0.361	-0.169/0.891	**0.182**
Δ CAL (mm)[4]	-1.431	-3.573/0.710	**0.191**	-0.612	-1.085/-0.138	**0.011**	-0.577	-1.470/0.314	0.206	0.234	-0.213/0.682	0.305
Δ BOP (%)[4]	-3.166	-6.627/0.303	**0.073**	-2.381	-3.362/-1.396	**<0.001**	-2.183	-3.413/-0.953	**<0.001**	0.358	-0.349/1.059	0.317

[1]β is the angular coefficient. [2]Categorical variables for which the first category is the reference. [3]Calcium intake at baseline before the intervention. **Bold:** *p*-value ≤ 0.20. [4]The difference between T2 and T0.
